# HuRInterDB: A Database Exploring the Landscape of lncRNA–Protein Interactions

**DOI:** 10.34133/csbj.0241

**Published:** 2026-10-21

**Authors:** Qianwen Xie, Wenju Sun, Jian Yan

**Affiliations:** ^1^ College of Life Sciences, Northwest University, Xi’an, China.; ^2^ Department of Biomedical Sciences, College of Biomedicine, City University of Hong Kong, Hong Kong SAR, China.; ^3^ Department of Urology, The First Affiliated Hospital of Xi’an Jiaotong University, Xi’an, China.

## Abstract

Long noncoding RNAs (lncRNAs) possess diverse biological functions and play key regulatory roles in biological processes by interacting with other biomolecules, such as proteins, RNAs, and the genome. High-throughput RNA-interaction experiments have generated large volumes of lncRNA–protein interaction records. The fragmented deposition of these records across disparate public databases complicates unified retrieval and integrative analysis. Here, we present HuRInterDB (Human lncRNA–Protein Interaction Database), a comprehensive and standardized database that focuses on human lncRNA–protein interactions. HuRInterDB is a user-friendly database designed to provide an integrated resource of lncRNA–protein interaction data from 621 high-throughput datasets (encompassing 2,440,594 lncRNA–protein interactions derived from 36,184 lncRNAs and 14,888 proteins) and to support functional research on lncRNA. The web interface supports interactive searching and visualization of lncRNA–protein associations. This curated resource streamlines integrative analysis of lncRNA–protein interactions and provides a standardized foundation for future computational and experimental studies. Distinctive features include an integrated analysis module that enables interactive visualization of protein–protein interaction networks, functional word clouds, and Gene Ontology enrichment bubble plots for any queried lncRNA, as well as download functionality. HuRInterDB is freely accessible at https://nwu26-hurinterdb.share.connect.posit.cloud/, with semiannual updates and complete data downloads.

## Introduction

Although the human genome is extensively transcribed, only approximately 2% of its RNA is protein-coding [1]. Long noncoding RNAs (lncRNAs), defined as transcripts longer than 200 nucleotides with limited coding potential, have emerged as key regulators of diverse biological processes [2,3]. Once dismissed as transcriptional noise, many lncRNAs are now recognized as functional participants in gene expression, translation, chromatin remodeling, genomic imprinting, alternative splicing, and signaling [4]. Their interactions with DNA, RNA, and proteins are pervasive and central to understanding the regulatory capacity of lncRNA in cellular processes [5,6]. For example, XIST interacts with PRC2 to influence its release and catalytic activation on the X chromosome in female mammals [7], while MALAT1 regulates alternative splicing through interactions with serine/arginine-rich (SR) splicing factors [8]. Accumulating evidence demonstrates that lncRNA–protein interactions constitute the primary molecular basis of lncRNA-mediated regulatory functions, making such interaction profiles indispensable for deciphering the biological roles of lncRNA. Notably, lncRNAs exhibit strong tissue and cell-type specificity in their expression and functional activities. Accordingly, integrating cell and tissue contextual information into lncRNA–protein interaction annotations is critical for accurately interpreting their context-dependent regulatory mechanisms. In addition, the dysregulation and cell-type-specific expression of lncRNA underlie their potential as disease biomarkers and therapeutic targets [9–11].

In recent years, considerable efforts have been devoted to characterizing human lncRNA–protein interactions across diverse biological contexts using multi-omics strategies [12]. RNA–protein interaction (RPI) data are derived from experimental evidence, literature mining, and computational predictions, with techniques such as pull-down assays, EMSA (electrophoretic mobility shift assay), FRET (fluorescence resonance energy transfer), and FISH (fluorescence in situ hybridization) used for validation. Recent advances in high-throughput experiments and computational methods have substantially improved the accuracy and coverage of RPI network construction and prediction. High-throughput experimental strategies can be broadly classified into protein- and RNA-centric approaches. Protein-centric methods, such as CLIP-seq (cross-linking and immunoprecipitation sequencing), eCLIP-seq (enhanced cross-linking and immunoprecipitation sequencing), LACE-seq (linear amplification of complementary DNA ends and sequencing), RIP-seq (RNA immunoprecipitation sequencing), and ARTR-seq (reverse transcription-based RBP binding sites sequencing), identify the RNAs associated with a given immunoprecipitated protein [13]. These methods typically rely on immunoprecipitation of the protein of interest followed by sequencing of associated RNAs, enabling transcriptome-wide identification of RNA-binding protein (RBP) targets at nucleotide resolution. Conversely, RNA-centric methods, such as RNA pulldown–MS (RNA pulldown assays coupled with mass spectrometry), ChIRP-MS (chromatin isolation by RNA purification followed by mass spectrometry), RAP-MS (RNA antisense purification-coupled with mass spectrometry), and CARPID (CRISPR-assisted RNA-protein interaction detection), isolate proteins associated with an RNA of interest, allowing unbiased discovery of proteins bound to a specific lncRNA. The recently developed PRIM-seq (protein-RNA interaction mapping by sequencing) enables concurrent *de novo* identification of RBPs and their associated RNAs in a global manner. It facilitates systematic discovery and prioritization of RBPs and their targets without requiring gene- or protein-specific reagents [14]. These advances have enabled the reconstruction of more comprehensive RPI networks. Nevertheless, the underlying data remain scattered across independent repositories, and this fragmentation creates a pressing need for systematic data integration.

Recognizing this need, several databases have been developed to catalog lncRNA interactions, including ENCORI (formerly starBase) [15], NPInter v5.0 [16], RNAInter v4.0 [17], and LncTarD 2.0 [18]. While valuable, these extensive RPI databases do not provide a coherent and comprehensive resource for lncRNA research. The critical gaps shared by existing resources can be summarized as follows: (a) limited scale: most databases contain only tens or hundreds of thousands of lncRNA–protein interactions; (b) lack of integrated analysis: while some databases offer basic search and download functions, none provides an integrated platform that seamlessly transitions from interaction query to downstream functional analysis, such as enrichment analysis; (c) limited visualization capabilities: for instance, generating publication-ready enrichment plots is typically possible only in standalone tools, requiring users to download interaction lists and import them into external software [e.g., Gene Ontology (GO) enrichment tools], which creates friction in the research workflow.

Driven by these critical research gaps and the rapidly growing interest in lncRNA studies, there is an urgent need to integrate newly emerging multi-omics evidence with diverse biological contextual information. To address this need, we conducted comprehensive curation and integration of lncRNA–protein interaction data and constructed an online resource termed HuRInterDB (Human lncRNA–Protein Interaction Database). This database leverages the aggregated datasets to facilitate functional research on human lncRNA. HuRInterDB is a comprehensive resource for human lncRNA–protein interactions, containing 2,440,594 interaction records derived from 36,184 lncRNA and 14,888 proteins. In addition to core search functions (query by lncRNA or protein) and data download options (full dataset or subsets), it offers an intuitive web toolkit supporting customizable analysis and visualization of lncRNA–protein interactions. In conclusion, HuRInterDB represents a valuable resource for lncRNA–protein interaction data and provides an effective data-mining platform for biological researchers.

## Materials and Methods

### Data collection and preprocessing

To construct a versatile database, we comprehensively collected public RNA-centric, protein-centric, and global RPI experimental datasets for HuRInterDB through extensive literature searches of the PubMed with keywords: “((RNA–protein interaction [Title/Abstract]) OR (RNA interaction [Title/Abstract]) OR (Protein inter-action [Title/Abstract]) OR (lncRNA Interaction [Title/Abstract]) OR (RBP lncRNA [Title/Abstract]))”.

These datasets were originally retrieved from public repositories, including GEO, ENCODE, and the ProteomeXchange Consortium. We restricted species to *Homo sapiens* and retained only lncRNA-relevant datasets (predominantly RNA-centric resources). Dataset selection followed the criteria below: (a) complete sample metadata are available; (b) samples were free from experimental perturbations including gene knockdown, gene knockout, or drug treatment; (c) peer-reviewed processed outputs are accessible, such as RNA lists, protein lists, or peak-call results. In this study, we collected pre-computed interaction results (peak tables and RBP-RNA binding lists) from published studies instead of re-analyzing raw sequencing or mass-spectrometry data. Only results with a *P* value < 0.05 were retained.

For each interaction entry, we compiled the associated lncRNA gene symbol, protein gene symbol, tissue- or cell-line context, experiment name, repository accession identifiers, and publication metadata (PubMed ID). To improve data interoperability, lncRNA and protein nomenclature were standardized using gene symbols to achieve consistency across heterogeneous source datasets. For mass-spectrometry-derived datasets, protein lists were adopted directly from the original publications. For sequencing-based datasets, lncRNA entries were directly extracted when the original study supplied a pre-defined gene list containing lncRNA. Where peak-call files were available, peaks were annotated against the GENCODE v49 lncRNA annotation file (gencode.v49.long_noncoding_RNAs.gtf), and lncRNA information was parsed from the resulting annotations. Peak files originating from the hg19 reference genome were converted to hg38 via liftOver prior to annotation. For differential gene expression analysis results, the threshold used was *P* < 0.05 and |logFC| > 1.

Initial data collection and preliminary processing were performed by one researcher. To minimize potential human bias and transcription errors, a random-sampling-based quality-control procedure was adopted to validate curated records. Three independent sampling rounds were carried out. Within each round, 50 datasets were randomly drawn from the full curated pool for independent cross-checking by a second researcher. Discrepancies uncovered during cross-checking were jointly evaluated by the 2 researchers; inconsistent entries were re-examined against the original literature or dataset files and corrected as appropriate. In its current version, a total of 621 omics datasets from 17 types of detection methods, sourced from peer-reviewed publications released between January 2012 and June 2026, were collected. Detailed metadata for each dataset were curated and provided in Table S1.

### Data integration and annotation

Information from all source datasets was aggregated. A de-redundancy step was subsequently performed: Entries sharing identical lncRNA, protein, and cell-line annotations were merged, while underlying individual supporting-evidence records were retained without removal.

To improve user-friendliness and enable smoother navigation, HuRInterDB incorporates external database hyperlinks for every data entry. These links allow users to explore supplementary lncRNA–protein interaction information outside our curated dataset, maximizing the utility of available public resources to support lncRNA functional studies. Specifically, each lncRNA entry contains a GeneCards hyperlink offering detailed transcript-level descriptions, including genomic context, expression profiles, and disease-related associations. Subcellular localization data for individual lncRNA are retrieved from RNALocate [19], a comprehensive resource for RNA subcellular localization. Structural information for interacting proteins is retrieved from InterPro [20]. Functional annotations and pathway assignments for interacting proteins are obtained from the Kyoto Encyclopedia of Genes and Genomes (KEGG) [21].

In addition, to maximize user interactivity, the database allows users to click any protein name within search results to fetch its full set of interacting lncRNA, which are rendered as an interactive network diagram. To preserve visual clarity, when the total number of interacting lncRNA exceeds the cutoff, only the top 200 lncRNAs sorted by interaction confidence score are displayed. This rounded threshold value of 200 is derived from the mean number (163.93) of RPIs.

### Calculation of interaction confidence scores

To quantitatively assess the reliability of individual RPI entries and mitigate intrinsic technical heterogeneity across diverse experimental assays, we built a multidimensional, evidence-weighted confidence-scoring system. This integrative confidence score was formulated with reference to prior studies [17,22] and incorporates 2 core dimensions: (a) trustworthiness of individual experimental evidence (*E*), which accounts for assay-level specificity and multiplicity of independent evidence, and (b) community-level consensus strength (*S*) for a given interaction across accessible public resources. Importantly, the interaction confidence score presented herein has not been rigorously benchmarked against gold-standard RPI datasets. It should thus be treated as a heuristic-ranking metric instead of an absolute quantitative measure reflecting the true reliability of reported interactions.

All experimental assays compiled in HuRInterDB were grouped into distinct evidence tiers based on their molecular resolution and capacity to capture direct physical binding [13,23–27]. Assays with high specificity that capture direct binding events [e.g., eCLIP-seq, PAR-CLIP (photoactivatable ribonucleoside-enhanced cross-linking and immunoprecipitation), and iCLIP-seq (individual-nucleotide resolution cross-linking and immunoprecipitation sequencing)] were allocated higher weights. By contrast, methods susceptible to indirect or proximity-driven associations (e.g., conventional RIP-seq) received relatively lower weights (Table 1).

**Table 1.** The weight distribution of each technology

Type	Method	Dataset number	Resolution	Interaction	*w*
RNA-centric	RNA pulldown–MS	20	Transcript	Direct + RNP	0.70
	ChIRP-MS	6	Transcript	Direct + RNP	0.75
	RAP-MS	6	Transcript	Direct + RNP	0.80
	HyPR-MS	3	Transcript	Direct + RNP	0.75
	CARPID	2	Transcript	Direct + RNP	0.80
	HPLC-MS	2	Transcript	Direct + RNP	0.70
	SILAC -MS	1	Transcript	Direct + RNP	0.70
	TREX	1	Transcript	Direct + RNP	0.80
Protein-centric	CLIP-seq	76	Near-nucleotide	Direct	0.90
	iCLIP-seq	5	Near-nucleotide	Direct	0.95
	eCLIP-seq	298	Near-nucleotide	Direct	1.00
	PAR-CLIP	46	Near-nucleotide	Direct	0.90
	HITS-CLIP	14	Near-nucleotide	Direct	0.85
	LACE-seq	2	Near-nucleotide	Direct	0.80
	RIP-seq	136	Transcript	Direct	0.75
	ARTR-seq	2	Near-nucleotide	Direct + RNP	0.80
Global	PRIM-seq	1	Transcript	Direct + RNP	0.75

Meanwhile, an interaction supported by a greater number of independent experimental assays is more likely to reflect a genuine biological association and thus warrants a higher confidence score. Nevertheless, a single high-throughput RPI study can generate thousands or even tens of thousands of putative interactions, which confers an elevated false-discovery risk compared with small-scale targeted experiments validating only a restricted set of interaction candidates. Accordingly, to accommodate dataset-level false-discovery risk inherent to high-throughput experiments, factor *S* imposes moderate weight reduction on interactions originating from datasets that produce substantial numbers of reported hits. The trustworthiness of experimental evidence (*E*) is computed as follows:

$$E=\sum_{i}\frac{w}{\log\left(n_{i}+1\right)}$$ (1)

where *i* denotes the index of each supporting dataset, *w* is the method-specific weight assigned to the corresponding assay, and *n_i_* represents the total number of reported interactions from the *i*th dataset.

The community-level trust metric (*S*) is quantitatively estimated from 2 bibliometric indicators retrieved from Google Scholar: citation count and publication year of the study reporting each interaction [28]. For each dataset-associated publication, we performed title-and-year-based queries to retrieve corresponding citation statistics. A higher citation count signifies broader recognition and validation of the finding by the research community, which contributes to an elevated confidence score. Google-Scholar-sourced citation values used for computing metric *S* and integrative confidence scores are refreshed every 6 months, synchronized with our database release cycle. New database releases incorporate up-to-date citation statistics to recalculate these confidence-related metrics. Specifically, metric *S* is computed as follows:

$$S=\sum_{i}\frac{\log\left(c_{i}+1\right)}{Y-y_{i}+1}$$ (2)

where ***c*** denotes the citation count of the corresponding publication, ***y*** is the publication year, and ***Y*** indicates the year of database update. For the current release, ***Y*** equals 2026.

These 2 metrics are combined to compute the integrative confidence score:

$$\text{Confidence Score}=\sqrt{E^{2}+S^{2}}$$ (3)

Interactions missing either the experimental-evidence component (*E* = 0, when *n_i_* = +∞, *i* = 1 and *w* = 0.7) or literature-support component (*S* = 0, which means no citation) are assigned a confidence score of 0 to avoid an artificially inflated score derived solely from the other remaining component. The raw composite score is subsequently normalized to a continuous scale spanning 0 to 1 to yield the final interaction confidence score. As a heuristic-ranking metric, this score quantitatively differentiates evidence strength across individual RPI entries and allows users to prioritize high-confidence interactions.

### Online RPI analysis module construction

The word-cloud plot and corresponding result table are derived from search results across the full dataset. Protein-name word frequencies were calculated by extracting protein information from these search outputs. The word cloud was then generated with the R package wordcloud2 (v0.2.1).

To generate high-resolution protein-binding profiles along lncRNA transcripts, we implemented the following workflow. First, lncRNA transcript-isoform annotations were retrieved from GENCODE v49. Second, eCLIP-seq peak files with detailed binding-site information were downloaded from the ENCODE portal and consolidated into a dedicated dataset. A peak was assigned to a given lncRNA if the entire peak interval lay within the gene boundaries of that lncRNA. For each valid peak, its binding center (midpoint coordinate) was computed from the peak’s start and end positions to facilitate visualization of protein-binding locations. Visualizations were produced using the R package ggplot2 (v4.0.3).

To explore the biological functions associated with protein, GO enrichment analysis was performed using the enrichGO() function from the clusterProfiler (v4.20.0) [29] with parameters ‘OrgDb = org.Hs.eg.db, ont = “ALL”, universe = all_protein, pAdjustMethod = “BH”, minGSSize = 10, maxGSSize = 500, pvalueCutoff = 1, qvalueCutoff = 0.05, readable = TRUE’. The analysis results were visualized using the R package enrichplot (v1.32.1).

Protein–protein interaction (PPI) networks were constructed via the getPPI() function from the clusterProfiler package (v4.20.0), with parameters ‘taxID = “9606”, add_nodes = 0, required_score = 700’ [30,31]. getPPI() retrieves PPI information from the STRING database (v12.0). The resulting networks were visualized using the ggtangle package (v0.1.3).

### Implementation

The HuRInterDB web portal is implemented in R Shiny and deployed on a cloud-based application hosting service for full accessibility. All source code was developed within RStudio. The front-end user interface is built with the R package shinydashboard (v0.7.3), and its layout is further customized using hypertext markup language (HTML) and cascading style sheets (CSS). Core R libraries powering the web application include DT (v0.34.0), shinyWidgets (v0.9.1), shinycssloaders (v1.1.0), data.table (v1.18.6), tidyverse (v2.0.0), igraph (v2.3.3), waiter (v2.5.1), and ggplot2 (v4.0.3).

The dataset generated by HuRInterDB is distributed under the Creative Commons Attribution 4.0 (CC-BY-4.0) license. All interactive tables in the web interface support searching, sorting, and on-demand downloading. In addition, the complete interaction dataset and corresponding metadata can be freely accessed through the Download page. This page also hosts 2 reference gene lists: the human transcription factor (TF) list retrieved from The Human Transcription Factors resource (https://humantfs.ccbr.utoronto.ca/allTFs.php) [32], and the RBP list obtained from RBPWorld (http://research.gzsys.org.cn/rbpworld/#/home) [33]. A full user tutorial for the web portal is provided on the About/Help page. A metadata dictionary is available to document every table field (Table S2), identifier category, and evidence- and confidence-score-related attributes.

## Results

### Overview of HuRInterDB

In recent years, a wide array of experimental approaches has been developed for systematic mapping of RPIs. To organize this growing body of data, we developed HuRInterDB as a specialized resource for curating and consolidating human lncRNA–protein interaction datasets. This resource enables researchers to leverage these data to gain mechanistic insights into lncRNA functions. HuRInterDB provides an intuitive web interface supporting querying, browsing, exploratory analysis, and visualization of interaction records. Users can perform flexible searches by combining any of 4 core parameters: (a) an lncRNA of interest, (b) a target protein (i.e., an RBP), (c) cell-type or tissue context, and (d) the experimental assay used for interaction detection. Search results are displayed in an interactive table equipped with sorting, filtering, and export functions, enabling users to customize outputs according to their specific research needs.

To construct a comprehensive, multifunctional lncRNA–protein interaction database, we performed systematic literature mining through keyword-based searches in PubMed to collect raw metadata. High-throughput sequencing and mass spectrometry datasets were retrieved from public repositories, including GEO [34], ENCODE, and the ProteomeXchange Consortium, with a data cutoff of June 2026. Only datasets explicitly reporting interactions involving human lncRNA were retained for downstream analysis (Fig. 1). Ultimately, we collected 17 categories of multi-omics data derived from 3 classes of detection strategies: RNA-centric, protein-centric, and global profiling (Table 1). To enhance data interoperability, all records were standardized into a unified format.

**Fig. 1.**
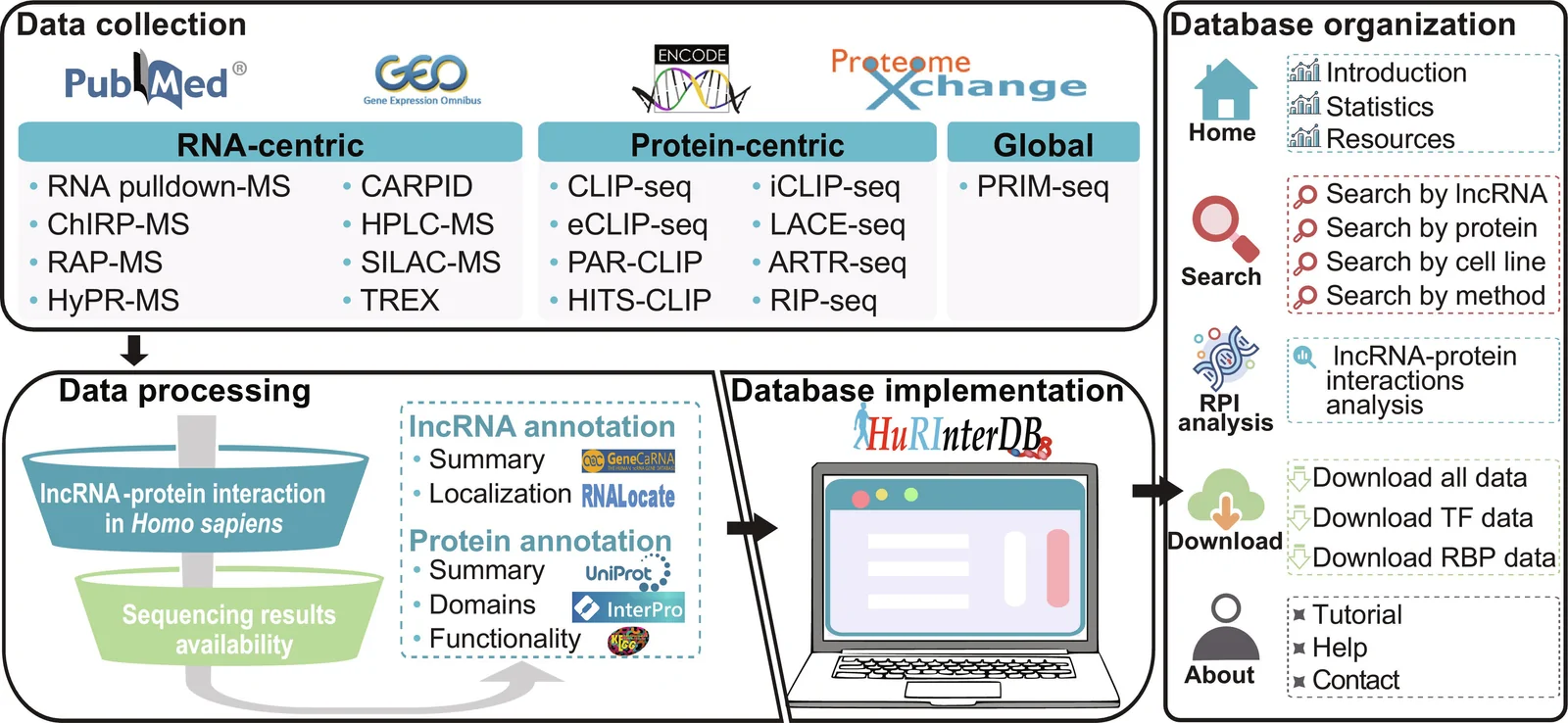
The overall workflow of HuRInterDB. LncRNA–protein interactions were derived from processed high-throughput datasets. Structural, subcellular-localization, and other functional annotations for the involved RNAs and proteins are mainly retrieved from publicly available external databases.

In its current release, HuRInterDB was constructed from experimental data sourced from 621 multi-omics datasets obtained from peer-reviewed publications (Table 1). These datasets span a broad range of experimental assays, including ChIRP-MS, CARPID, RIP-seq, CLIP-seq, and PRIM-seq, which represent major technological platforms for mapping RPIs. Following systematic integration of these resources, the current version of HuRInterDB contains 2,440,594 lncRNA–protein interaction records, corresponding to 36,184 unique lncRNA genes and 14,888 unique proteins. On average, each lncRNA has 67.45 binding partners, while each protein interacts with 163.93 lncRNA. Each interaction entry is annotated with comprehensive metadata: (a) cell-line or tissue context, (b) experimental detection assay, and (c) a direct hyperlink to the original raw data source.

### Searching and querying data in HuRInterDB

To assist the research community in exploring and analyzing lncRNA–protein interaction data, HuRInterDB provides a comprehensive and intuitive web interface that supports conditional queries, exploratory analysis, visualization, and data download. The overall user interface of HuRInterDB is illustrated in Fig. 2 and comprises 5 major functional modules: (a) Home, (b) Search, (c) RPI analysis, (d) Download, and (e) About.

**Fig. 2.**
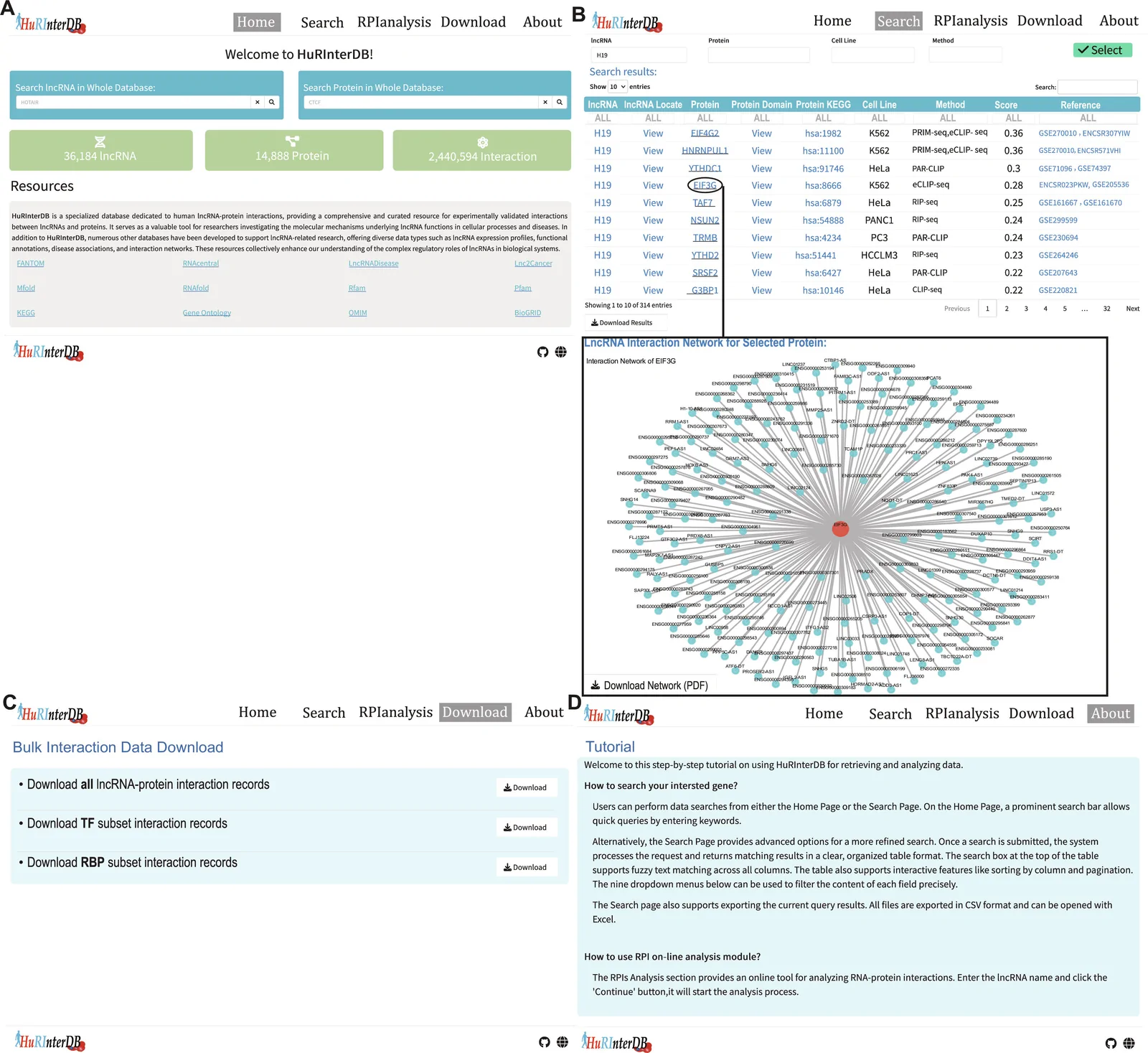
HuRInterDB web interface. (A) “Home” interface of HuRInterDB; a search panel exists at the top of the page, with search options grouped into 2 categories. It also includes brief statistical information about HuRInterDB and useful resources. (B) The options available on “Search” page include searching by lncRNA, protein, cell line/tissue, and method. The results of a search are presented in an interactive table that can be further filtered using the column filters below the labels. By clicking on a protein name in the results list, users can view all lncRNAs that interact with that protein. (C) Options available on the “Download” section. Users can download all RPI information or only download RPI about TF/RBP. (D) Options available on the “About” page.

#### Home page

The Home page provides a general overview of the database (Fig. 2A). Two separate search modes, “Search by lncRNA” and “Search by protein”, are available through panels located at the top of the Home page. The “Search by lncRNA” function allows users to filter by a chosen lncRNA and returns its associated RPI entries. Conversely, “Search by protein” retrieves all RPI records corresponding to a selected protein. Clicking the search icon redirects users to the dedicated Search page, where query results are displayed in a paginated interactive table below the search panel. Summary statistics of database contents, including total counts of datasets, RPI, and lncRNA, are shown in the central section of the Home page. In addition, the Home page offers direct links to external lncRNA-related resources, including RNAcentral [35], LncRNADisease [36], Lnc2Cancer [37], Mfold [38], RNAfold, and Rfam [39].

#### Search page

The Search module provides an efficient interactive platform that supports various search operations for data querying and subset generation (Fig. 2B). Within this module, users can browse data by selecting a target lncRNA, protein, cell line, or experimental method from drop-down selection boxes. After filtering, an intuitive tabular result list is displayed immediately on the same page based on the selected identifiers. Search results contain comprehensive RPI information: lncRNA name, binding partner protein name, cell type or tissue origin, experimental assay, and associated annotation details.

Leveraging its abundant cross-reference resources, HuRInterDB connects to GeneCards through hyperlinks embedded within lncRNA entries. Additional molecular annotations for lncRNA and proteins are appended to every interaction entry: lncRNA subcellular localization retrieved from RNALocate [19], protein domain information obtained from InterPro [20], and protein functional annotations sourced from KEGG [21]. This integration enables users to seamlessly cross-reference HuRInterDB records with external resources. A direct source hyperlink for each entry is also provided in the result table. Sorting and filtering of outputs can be performed using search fields beneath column headers. In addition, users can download interaction data by clicking the “Download Results” button at the bottom of the detail page. To enhance interactivity, clicking any protein name in the result table retrieves the full set of lncRNAs that interact with that protein, and the corresponding interaction network is rendered as a graph. For convenient data sharing and figure preparation, users can click the download button to export this interaction network diagram to their local machine.

#### RPI analysis page

HuRInterDB features an interactive analysis module that enables users to identify and visualize proteins interacting with a given lncRNA by entering the lncRNA name in the input box (Fig. 3). After clicking the “Continue” button, analytical outputs are generated as a word cloud plot, a PPI network, and a GO enrichment dot plot. A complete list of binding proteins is shown below the visualization panel. This functionality helps users systematically explore the biological functions and mechanistic features of the queried lncRNA. Detailed descriptions of these online analytical tools are provided in the following sections.

**Fig. 3.**
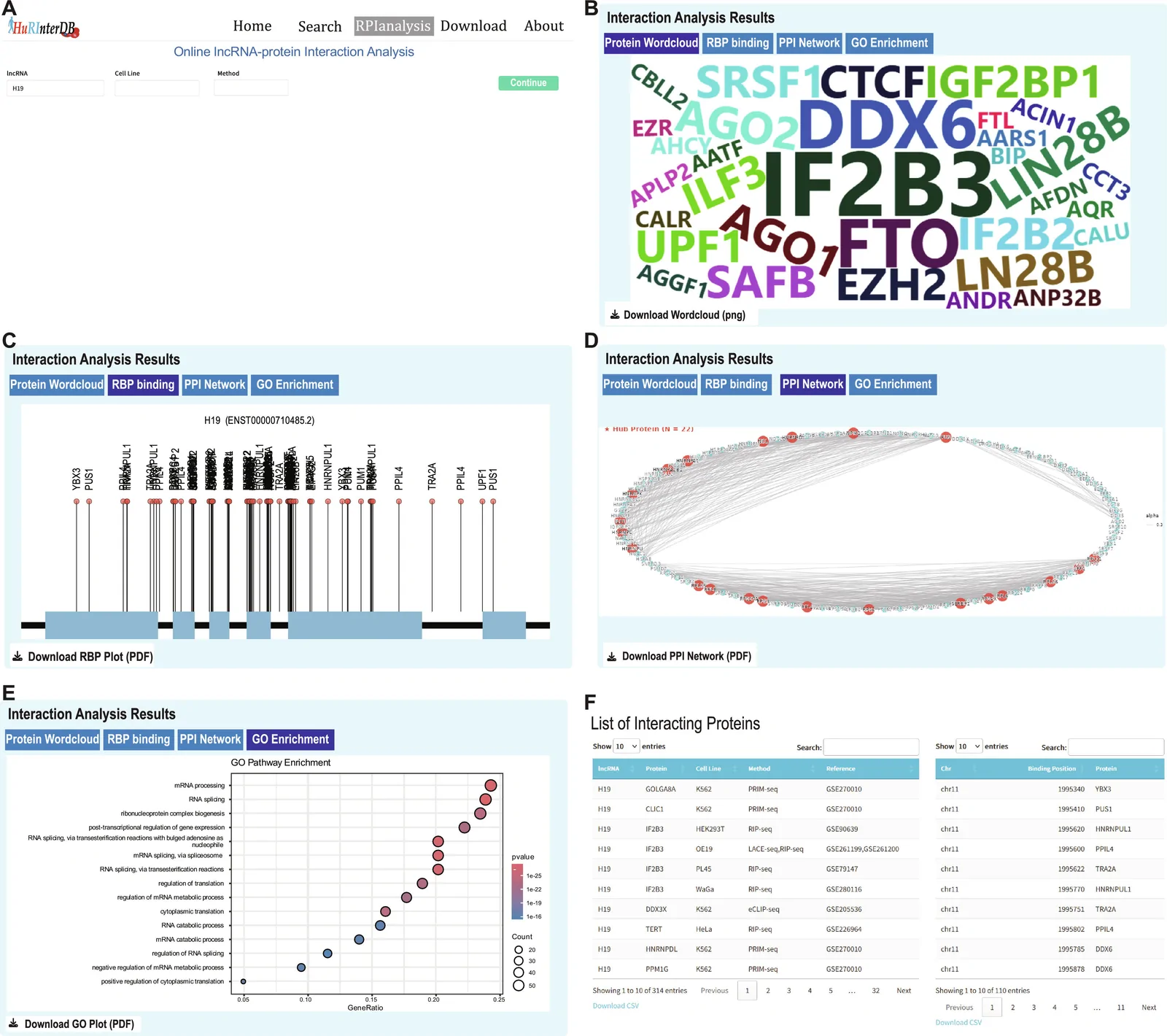
Online analysis of lncRNA–protein interaction offered by HuRInterDB and an example. (A) Users type the name of the lncRNA of interest into the input box. Upon clicking the “Continue” button, analysis results appear in the central region of the page. These outputs comprise a word cloud (B) offering an overview of the interacting-protein atlas, a schematic plot (C) depicting protein-binding positions across the full-length lncRNA, physical protein–protein interactions (D) among these binding proteins, and a dot plot visualizing GO enrichment result computed (E). *P* values are calculated using the hypergeometric distribution, and the Benjamini–Hochberg method is applied for multiple-testing correction. For the enrichment dot plot, the *x* axis represents GeneRatio, the proportion of query proteins annotated to a given GO term; the *y* axis displays GO-term names ordered by adjusted *P* value; bubble size corresponds to the number of query proteins assigned to each GO term; and bubble color indicates adjusted *P* values. Furthermore, a tabular view at the bottom of the page provides a complete protein list (F, left) as well as genomic protein-binding sites (F, right).

#### Download page

HuRInterDB provides unrestricted open access to all lncRNA–protein interaction records through the Download page. To accommodate diverse research needs, this page offers flexible download options: Users can obtain the complete interaction repository as a single consolidated file or export filtered subsets restricted to interactions involving selected RBPs or TFs (Fig. 2C).

#### About page

To facilitate cross-page navigation and help users interpret output results, a comprehensive user tutorial is available under the “About” menu (Fig. 2D). Most common issues encountered during routine database usage can be quickly resolved by consulting this documentation.

### An example of online RPI analysis

LncRNAs have emerged as critical regulators across a wide spectrum of biological processes and represent promising therapeutic targets for human diseases. Accordingly, deciphering lncRNA functions and their underlying regulatory mechanisms is essential for identifying novel clinical therapeutic targets. Because most lncRNAs interact with a large repertoire of proteins, visualizing these interaction networks is indispensable for deriving biologically meaningful insights. Intuitive graphical representations enable users to readily identify hub proteins, uncover functional modules, and prioritize candidate molecules for downstream experimental validation. To address this need, we implemented the RPI analysis module, a simple yet user-friendly online tool dedicated to the interactive visualization and analysis of lncRNA–protein interaction datasets. Users can navigate to the RPI analysis page directly via the dedicated button on the navigation bar. For any user-defined lncRNA, this module generates 4 distinct visualizations based on the collection of binding proteins associated with it: (a) a protein word cloud, (b) an RBP-binding schematic diagram, (c) a PPI network, and (d) a GO enrichment bubble plot (Fig. 3). All generated outputs can be downloaded locally. Collectively, these visualizations provide a comprehensive, multi-perspective view of the functional landscape associated with the queried lncRNA.

Users can type a target lncRNA in the input box (Fig. 3A). Upon clicking the “Continue” button, the RPI analysis tool renders the corresponding outputs as visualizations and displays detailed interaction records in a table at the bottom of the page (Fig. 3F). In addition, cell line and experimental method filter options allow users to perform more focused, context-specific analyses. The result visualization panel consists of 4 separate tabs: “Protein Word Cloud”, “RBP Binding”, “PPI”, and “GO Enrichment”. Each tab hosts its respective analytical outputs, which users can view by switching to the tab of interest.

After the user clicks the “Continue” button, the RPI analysis tool identifies proteins that interact with the target lncRNA and counts the frequency of each protein. The results are then presented as a word cloud (Fig. 3B). Word clouds provide a visual and intuitive summary of protein interaction data, in which proteins detected more frequently stand out with larger font sizes. This allows users to rapidly identify key proteins and potential functional themes, making word clouds a valuable tool for initial hypothesis generation prior to more in-depth analysis. As an example, some of the proteins that interact with lncRNA H19, as displayed in the word cloud, have been validated by multiple studies, such as EZH2 [40–42] and CTCF [43,44] (Fig. 3B). However, the mechanisms by which most of these proteins interact with H19 remain unclear. Another illustrative example involves MEG3, an lncRNA known to promote the degradation of SHP (small heterodimer partner) mRNA via interaction with PTBP1 [45]. This previously documented regulatory relationship is recapitulated in our word cloud (Fig. S1A). Nevertheless, one important caveat must be emphasized: The word cloud output is susceptible to experimental ascertainment bias. Over-studied proteins may achieve high visual prominence simply due to more available published records, rather than reflecting a genuine biological preference for this lncRNA–protein interaction.

Protein-centric experimental datasets yield not only RPI pairs but also relatively fine-grained binding-site information. For example, eCLIP-seq assays identify discrete binding footprints (peaks), typically approximately 50 base pairs in width, which pinpoint where RBPs physically contact RNA molecules. To enable higher-resolution characterization of lncRNA–protein interactions, our web tool utilizes high-quality eCLIP-seq datasets curated from the ENCODE resource to generate a schematic map illustrating RBP binding profiles along the entire length of a user-queried lncRNA transcript (Fig. 3C). This visualization helps researchers inspect the spatial distribution of individual binding events and facilitates the biological interpretation of these interaction data. Users can manually switch among available transcript isoforms and select alternative transcripts for visualization based on their research objectives. As exemplified by the H19 transcript in Fig. 3C, a substantial number of RBP binding peaks cluster around splice-site regions, suggesting potential functional involvement of these proteins in splicing-related regulatory processes. If no results are available, the diagram is displayed as blank (Fig. S1B).

To identify hub proteins and dissect the higher-order architecture of lncRNA-associated protein complexes, this tool constructs PPI networks for proteins that bind a queried lncRNA (Fig. 3D and Fig. S1C). Hub proteins, defined as nodes with the highest connectivity degree, are marked in red for easy visual identification. By mapping associations among these binding proteins, PPI networks provide a systems-level view of cellular processes linked to the queried lncRNA. Instead of evaluating each binding protein in isolation, this approach reveals how these proteins operate collectively within multi-component biological assemblies, including signaling cascades, transcriptional regulatory circuits, and chromatin-remodeling complexes. This systems-oriented perspective allows researchers to go beyond conventional single-protein investigations and facilitates the detection of emergent properties such as functional modules, hub nodes, and pathway crosstalk that would otherwise remain unrecognized.

To further dissect the biological functions of lncRNA, users can perform GO enrichment analysis on their interacting partner proteins. This functional profiling helps infer potential cellular roles for individual lncRNA by leveraging the annotated molecular functions, biological processes, and cellular components of their binding protein set. GO enrichment analysis is implemented using the hypergeometric test followed by Benjamini–Hochberg FDR (false discovery rate) correction (Fig. 3E and Fig. S1D). When this analytical workflow was applied to H19-interacting proteins, enrichment signals pointed to widespread involvement in RNA splicing. Additional prominent functional categories included mRNA processing, mRNA metabolism, and translation (Fig. 3E). These computational observations are consistent with previously reported experimental evidence. For example, H19 drives colorectal cancer progression by recruiting splicing factors RBFOX2 and hnRNPM to RAC1, thereby modulating the alternative splicing of RAC1 transcripts [46]. In another reported regulatory mechanism, H19 represses translation by blocking the association between eIF4A2 and PINK1 mRNA [47]. Consistent with previous findings that MEG3 promotes SHP mRNA degradation [45], GO enrichment analysis further indicates that MEG3 plays a role in biological processes related to mRNA processing.

Users can export multiple types of output generated by the interactive analysis module, including all rendered visualizations, the full list of candidate proteins, and comprehensive data on protein binding positions (Fig. 3F). These downloadable resources enable end users to reuse the analytical outputs for downstream custom analyses and obviate the need for additional manual data processing.

### Comparison with existing resources

Over the past decade, the rapid accumulation of high-throughput RPI data has spurred the development of numerous specialized databases, reflecting growing recognition of the functional importance of RPIs. These resources have become indispensable for studying post-transcriptional regulation, yet they often fall into 2 categories: those with a broad, multi-RNA scope (e.g., RNAInter) and those dedicated to specific regulatory axes (e.g., ENCORI, which focuses primarily on RNA–RNA interactions). To rigorously assess the distinct contributions of HuRInterDB within this evolving landscape and to quantify its advances over current state-of-the-art resources, we performed a systematic comparison with 3 representative, widely adopted databases: ENCORI, NPInter, and RNAInter (Table 2).

**Table 2.** Comparison with other major interaction databases

Feature	HuRInterDB	ENCORI	NPInter v5.0	RNAInter v4.0
Unique interactions ^a^	2,008,724	176,142	488,295	3,005,054
Unique lncRNA ^a^	36,184	13,380	59,847	23,908
Unique proteins ^a^	14,888	282	1,211	8,703
RNA-centric data	✓	✗	✓	✓
Global data (PRIM-seq)	✓	✗	✗	✗
Word cloud generation	✓	✗	✗	✗
RBP binding schematic	✓	✗	✗	✗
PPI network	✓	✗	✗	✓
GO enrichment	✓	✗	✗	✗
References	This study	[15]	[16]	[17]
Released in	2026	2026	2023	2022

^a^ Unique interactions refer to distinct human lncRNA–protein pairs. All statistical results exclude computationally predicted interactions. Comparable statistical metrics for other databases were computed using datasets retrieved from their respective download pages. Unique interactions are quantified as nonredundant lncRNA–protein pairs. The counts of lncRNA and proteins correspond to the numbers of distinct, nonredundant lncRNA and protein identifiers, respectively.

#### Data scale comparison

Compared with representative public databases, HuRInterDB offers broader coverage of lncRNA–protein interactions and a larger overall dataset scale than several existing resources. For reference, ENCORI comprises 176,142 RPI entries from 13,380 lncRNA; NPInter covers 488,295 lncRNA–protein interactions from 59,847 lncRNA; and RNAInter contains 3,005,054 RPI entries derived from 23,908 lncRNA. Using the lncRNA H19 as a case study, HuRInterDB contains 292 unique proteins that interact with H19, surpassing most existing databases in coverage (Fig. S1E and Table S3), including ENCORI (*n* = 33), NPInter (*n* = 64), and RNAInter (*n* = 247).

#### Inclusion of recent data and contextual annotations

HuRInterDB incorporates recently emerged data modalities, such as PRIM-seq, which are absent from other RPI databases. Moreover, each interaction entry in HuRInterDB is annotated with corresponding cell line source metadata, a feature largely lacking in other RPI resources. This contextual information is of substantial biological importance: RNA-driven binding events are highly dynamic and can exhibit divergent binding profiles shaped by the cellular microenvironment. In addition, lncRNA expression levels vary considerably across tissues and cell types [48,49]; awareness of these expression patterns is indispensable for accurate functional interpretation of lncRNA. Beyond cell line metadata, each entry in HuRInterDB is further enriched with comprehensive auxiliary annotations, encompassing the subcellular localization of lncRNA as well as functional domain information for their interacting partner proteins.

#### Specialized online tools

HuRInterDB provides a full suite of online utilities for the analysis and visualization of lncRNA-interacting proteins. Among publicly available counterpart databases, only NPInter supports visualization of lncRNA–protein interaction networks; most other databases completely lack built-in analytical and visualization modules. By converting complex interaction datasets into intuitive graphical outputs (including PPI networks, RBP-binding schematics, word clouds, and enrichment bubble plots), HuRInterDB addresses the limitations inherent in plain tabular data formats. These interactive visualizations substantially lower barriers for users exploring lncRNA-related biological functions. This tool suite enables on-the-fly online characterization of molecular features for proteins that interact with a user-defined lncRNA. Such integrated, interactive analytical functionality is unavailable in comparable resources.

#### Database scope and focus

Unlike other comparable public databases, HuRInterDB is specifically designed for curating high-quality, annotated human lncRNA–protein interactions. While these multi-purpose databases offer considerable value by compiling diverse classes of nucleic acid–protein interactions, they are not optimized for the distinct biological characteristics of lncRNA. This broad, general-purpose scope often diminishes their practical utility for lncRNA-focused research.

Furthermore, HuRInterDB supports cross-platform usage. Users can either access the web portal remotely from various devices or deploy the resource locally on personal computers for offline analysis. Collectively, these features enable researchers to rapidly generate testable biological hypotheses by integrating high-quality interaction datasets with built-in analytical and visualization functionalities. Consequently, HuRInterDB facilitates advances in the systematic functional annotation of lncRNA.

## Discussion

LncRNAs regulate numerous critical biological processes, including gene expression, chromatin remodeling, and signal transduction. Consequently, they are essential for understanding the molecular basis of a wide range of human diseases [50,51]. The recent surge of interest in lncRNA functional studies has generated a vast and diverse landscape of multi-omics data, encompassing transcriptomics, proteomics, and interactomics. However, as sequencing technologies continue to advance and public repositories accumulate ever-larger datasets, the efficient and systematic integration and analysis of these heterogeneous multi-omics data have become a daunting task for researchers. To address this challenge, we manually curated sequencing data from 621 published omics datasets and established HuRInterDB, a relational database specialized for collecting and curating RPIs involving human lncRNA. HuRInterDB integrates data across multiple experimental modalities, including CLIP-seq, RIP-seq, ChIRP-MS, and PRIM-seq. In addition, HuRInterDB provides an interactive tool for the analysis and visualization of RPIs, offering users novel biological insights. Overall, HuRInterDB aims to serve as a comprehensive web-based mining platform for researchers, providing a valuable resource for fundamental studies of lncRNA and beyond.

Recent advances in RPI modeling, exemplified by LPItabformer [52], BioPrediction-RPI [53], and RIscoper 2.0 [54], have highlighted the need for high-quality, standardized interaction datasets to train and assess predictive frameworks. In addition to supporting conventional biological analyses of lncRNA–protein interactions, HuRInterDB serves as a valuable resource for machine-learning-driven RPI research through its standardized, machine-readable data exports.

Despite these strengths, HuRInterDB has several inherent limitations. The quality of curated entries fundamentally depends on the original experimental studies from which the data were obtained. In addition, manual curation is inherently time-consuming and may be subject to subjective judgment or human error, which could affect the consistency and completeness of the database. Although we adopted a unified processing pipeline and strict curation criteria, users should consider the original detection techniques when interpreting results, given that distinct experimental methods differ in false-positive rates and technical resolution. Full-scale ROC (receiver operating characteristic)-driven validation of the confidence score is hindered by the limited availability of gold-standard lncRNA–protein interaction datasets for large-scale benchmarking. We recognize this as an important limitation of HuRInterDB, and future improvements to the scoring framework will be pursued as additional high-confidence reference interaction data accumulate. Furthermore, expression levels of lncRNA and their protein-binding partners could improve the performance of the interaction confidence scoring metric; however, full gene expression profiles are not available for some cell lines covered in the current database. In future work, we plan to integrate such expression-derived information to refine our interaction scoring framework.

Future developments will focus on incorporating additional high-quality datasets to enhance the overall performance and scientific utility of HuRInterDB. Specifically, we intend to perform semiannual database updates through systematic screening of newly published literature and recently released high-throughput datasets.

In summary, HuRInterDB is a novel, comprehensive resource for investigating RPIs and represents an important advance in lncRNA research. As a dedicated resource for exploring the lncRNA–protein interactome, it extends the capabilities of existing general-purpose or narrowly focused databases. We expect HuRInterDB to facilitate deeper mechanistic insights into RPI, accelerate translational research, and ultimately aid the development of innovative therapies addressing unmet medical needs across multiple human diseases.

## Data Availability

All datasets analyzed in this study were obtained from publicly available sources. The source code and data underlying this article are available on github at (https://github.com/NWU26/HuRInterDB/).

## References

[1] ENCODE Project Consortium. An integrated encyclopedia of DNA elements in the human genome. Nature. 2012;489(7414):57–74. 10.1038/nature11247 PMC3439153 22955616

[2] Mattick JS, Amaral PP, Carninci P, Carpenter S, Chang HY, Chen L-L, Chen R, Dean C, Dinger ME, Fitzgerald KA, et al. Long non-coding RNAs: Definitions, functions, challenges and recommendations. Nat Rev Mol Cell Biol. 2023(6):430–447. 10.1038/s41580-022-00566-8 PMC10213152 36596869

[3] Chen L-L, Kim VN. Small and long non-coding RNAs: Past, present, and future. Cell. 2024;187(23):6451–6485. 10.1016/j.cell.2024.10.024 39547208

[4] Kopp F, Mendell JT. Functional classification and experimental dissection of long noncoding RNAs. Cell. 2018;172(3):393–407. 10.1016/j.cell.2018.01.011 PMC5978744 29373828

[5] Licatalosi DD, Darnell RB. RNA processing and its regulation: Global insights into biological networks. Nat Rev Genet. 2010;11(1):75–87. 10.1038/nrg2673 PMC3229837 20019688

[6] Müller-McNicoll M, Neugebauer KM. How cells get the message: Dynamic assembly and function of mRNA-protein complexes. Nat Rev Genet. 2013;14(4):275–287. 10.1038/nrg3434 23478349

[7] Lee YW, Weissbein U, Blum R, Lee JT. G-quadruplex folding in Xist RNA antagonizes PRC2 activity for stepwise regulation of X chromosome inactivation. Mol Cell. 2024;84(10):1870–1885.e9. 10.1016/j.molcel.2024.04.015 PMC11505738 38759625

[8] Tripathi V, Ellis JD, Shen Z, Song DY, Pan Q, Watt AT, Freier SM, Bennett CF, Sharma A, Bubulya PA, et al. The nuclear-retained noncoding RNA MALAT1 regulates alternative splicing by modulating SR splicing factor phosphorylation. Mol Cell. 2010;39(6):925–938. 10.1016/j.molcel.2010.08.011 PMC4158944 20797886

[9] Esteller M. Non-coding RNAs in human disease. Nat Rev Genet. 2011;12(12):861–874. 10.1038/nrg3074 22094949

[10] Pereira B, Billaud M, Almeida R. RNA-binding proteins in cancer: Old players and new actors. Trends Cancer. 2017;3(3):506–528. 10.1016/j.trecan.2017.05.003 28718405

[11] Liu K, Gao L, Ma X, Huang J-J, Chen J, Zeng L, Ashby CR Jr, Zou C, Chen ZS. Long non-coding RNAs regulate drug resistance in cancer. Mol Cancer. 2020;19(1):54. 10.1186/s12943-020-01162-0 PMC7066752 32164712

[12] Van Nostrand EL, Freese P, Pratt GA, Wang X, Wei X, Xiao R, Blue SM, Chen JY, Cody NA, Dominguez D, et al. A large-scale binding and functional map of human RNA-binding proteins. Nature. 2020;583(7818):711–719. 10.1038/s41586-020-2077-3 PMC7410833 32728246

[13] Yi W, Yan J. Decoding RNA–protein interactions: Methodological advances and emerging challenges. Adv Genet. 2025;6(2):2500011. 10.1002/ggn2.202500011 PMC12245534 40657556

[14] Qi Z, Xue S, Chen J, Zhao W, Johnson K, Wen X, Charles Richard JL, Lin P, Zhong S. Genome-wide mapping of RNA-protein associations through sequencing. Nat Biotechnol. 2025;44(7):1202–1212. 10.1038/s41587-025-02780-z 40925998

[15] Zhou K, Huang J, Liu S, Zheng W, Liu S, Xing Y, Cao Y, Chen Y, Hu X, Cai L, et al. An encyclopedic regulatory and functional atlas of RNA interactomes. Nat Methods. 2026;23(6):1109–1114. 10.1038/s41592-026-03105-x 42185542

[16] Zheng Y, Luo H, Teng X, Hao X, Yan X, Tang Y, Zhang W, Wang Y, Zhang P, Li Y, et al. NPInter v5.0: ncRNA interaction database in a new era. Nucleic Acids Res. 2023;51(D1):D232–D239. 10.1093/nar/gkac1002 PMC9825547 36373614

[17] Kang J, Tang Q, He J, Li L, Yang N, Yu S, Wang M, Zhang Y, Lin J, Cui T, et al. RNAInter v4.0: RNA interactome repository with redefined confidence scoring system and improved accessibility. Nucleic Acids Res. 2022;50(D1):D326–D332. 10.1093/nar/gkab997 PMC8728132 34718726

[18] Zhao H, Yin X, Xu H, Liu K, Liu W, Wang L, Zhang C, Bo L, Lan X, Lin S, et al. LncTarD 2.0: An updated comprehensive database for experimentally-supported functional lncRNA–target regulations in human diseases. Nucleic Acids Res. 2023;51(D1):D199–D207. 10.1093/nar/gkac984 PMC9825480 36321659

[19] Wu L, Wang L, Hu S, Tang G, Chen J, Yi Y, Xie H, Lin J, Wang M, Wang D, et al. RNALocate v3.0: Advancing the repository of RNA subcellular localization with dynamic analysis and prediction. Nucleic Acids Res. 2025;53(D1):D284–D292. 10.1093/nar/gkae872 PMC11701552 39404071

[20] Blum M, Andreeva A, Florentino LC, Chuguransky SR, Grego T, Hobbs E, Pinto BL, Orr A, Paysan-Lafosse T, Ponamareva I, et al. InterPro: The protein sequence classification resource in 2025. Nucleic Acids Res. 2025;53(D1):D444–D456. 10.1093/nar/gkae1082 PMC11701551 39565202

[21] Kanehisa M, Furumichi M, Sato Y, Kawashima M, Ishiguro-Watanabe M. KEGG for taxonomy-based analysis of pathways and genomes. Nucleic Acids Res. 2023;51(D1):D587–D592. 10.1093/nar/gkac963 PMC9825424 36300620

[22] Li T, Wernersson R, Hansen RB, Horn H, Mercer J, Slodkowicz G, Workman CT, Rigina O, Rapacki K, Stærfeldt HH, et al. A scored human protein–protein interaction network to catalyze genomic interpretation. Nat Methods. 2017;14(1):61–64. 10.1038/nmeth.4083 PMC5839635 27892958

[23] Sahadevan S, Sekaran T, Hentze MW, Perez-Perri JI, Schwarzl T. Signal or noise? RNA-binding proteins and the challenges of binding site assignments. Nucleic Acids Res. 2026;54(9):gkag452. 10.1093/nar/gkag452 PMC13171450 42130068

[24] Lundrigan E, Evers P, Scott E, Pezacki JP. Unnatural amino acid and emerging chemistry approaches to map RNA–protein interactions. Angew Chem Int Ed Engl. 2026;65(6): Article e20744. 10.1002/anie.202520744 PMC12865153 41388880

[25] Régis M, Pulcina P, Kretov DA. Decoding RNA-protein interactions using high-throughput methods. RNA Biol. 2026;23(1):1–21. 10.1080/15476286.2026.2623240 PMC12915805 41605250

[26] Chakrabarty S, Roy S, Sarkar S, Singh KK. RNA-protein interaction techniques - a historical and comparative analysis. Biochim Biophys Acta Gene Regul Mech. 2025;1868(4): Article 195115. 10.1016/j.bbagrm.2025.195115 40945904

[27] Hafner M, Katsantoni M, Köster T, Marks J, Mukherjee J, Staiger D, Ule J, Zavolan M. CLIP and complementary methods. Nat Rev Methods Primers. 2021;1(1):20.

[28] Dunaiski M, Geldenhuys J, Visser W. On the interplay between normalisation, bias, and performance of paper impact metrics. J Informetr. 2019;13(1):270–290.

[29] Wu T, Hu E, Xu S, Chen M, Guo P, Dai Z, Feng T, Zhou L, Tang W, Zhan LI, et al. clusterProfiler 4.0: A universal enrichment tool for interpreting omics data. The. Innovation. 2021;2(3): Article 100141. 10.1016/j.xinn.2021.100141 PMC8454663 34557778

[30] Bhattacharjee N, Peela SCM, Halder A, Mathew B, Samanta S, Gujral S, Kumari S, Panda S, Ganguly R, Ahuja G, et al. Gene dependency-informed inference of response to targeted cancer therapies. Nat Commun. 2026;17:7251. 10.1038/s41467-026-73977-2 PMC13396380 42259811

[31] Saar KL, Scrutton RM, Bloznelyte K, Morgunov AS, Good LL, Lee AA, Teichmann SA, Knowles TP. Protein condensate atlas from predictive models of heteromolecular condensate composition. Nat Commun. 2024;15(1):5418. 10.1038/s41467-024-48496-7 PMC11237133 38987300

[32] Lambert SA, Jolma A, Campitelli LF, Das PK, Yin Y, Albu M, Chen X, Taipale J, Hughes TR, Weirauch MT. The human transcription factors. Cell. 2018;172(4):650–665. 10.1016/j.cell.2018.01.029 PMC12908702 29425488

[33] Liao J-Y, Yang B, Shi C-P, Deng W-X, Deng J-S, Cen M-F, Zheng BQ, Zhan ZL, Liang QL, Wang JE, et al. RBPWorld for exploring functions and disease associations of RNA-binding proteins across species. Nucleic Acids Res. 2025;53(D1):D220–D232. 10.1093/nar/gkae1028 PMC11701580 39498484

[34] Barrett T, Wilhite SE, Ledoux P, Evangelista C, Kim IF, Tomashevsky M, Marshall KA, Phillippy KH, Sherman PM, Holko M, et al. NCBI GEO: Archive for functional genomics data sets—Update. Nucleic Acids Res. 2013;41(D1):D991–D995. 10.1093/nar/gks1193 PMC3531084 23193258

[35] RNAcentral Consortium. RNAcentral in 2026: Genes and literature integration. Nucleic Acids Res. 2026;54(D1):D303–D313. 10.1093/nar/gkaf1329 PMC12807676 41404707

[36] Lin X, Lu Y, Zhang C, Cui Q, Tang Y-D, Ji X, Cui C. LncRNADisease v3.0: An updated database of long non-coding RNA-associated diseases. Nucleic Acids Res. 2024;52(D1):D1365–D1369. 10.1093/nar/gkad828 PMC10767967 37819033

[37] Gao Y, Shang S, Guo S, Li X, Zhou H, Liu H, Sun Y, Wang J, Wang P, Zhi H, et al. Lnc2Cancer 3.0: An updated resource for experimentally supported lncRNA/circRNA cancer associations and web tools based on RNA-seq and scRNA-seq data. Nucleic Acids Res. 2021;49(D1):D1251–D1258. 10.1093/nar/gkaa1006 PMC7779028 33219685

[38] Zuker M. Mfold web server for nucleic acid folding and hybridization prediction. Nucleic Acids Res. 2003;31(13):3406–3415. 10.1093/nar/gkg595 PMC169194 12824337

[39] Ontiveros-Palacios N, Cooke E, Nawrocki EP, Triebel S, Marz M, Rivas E, Griffiths-Jones S, Petrov AI, Bateman A, Sweeney B. Rfam 15: RNA families database in 2025. Nucleic Acids Res. 2025;53(D1):D258–D267. 10.1093/nar/gkae1023 PMC11701678 39526405

[40] Luo M, Li Z, Wang W, Zeng Y, Liu Z, Qiu J. Long non-coding RNA H19 increases bladder cancer metastasis by associating with EZH2 and inhibiting E-cadherin expression. Cancer Lett. 2013;333(2):213–221. 10.1016/j.canlet.2013.01.033 23354591

[41] Singh N, Ramnarine VR, Song JH, Pandey R, Padi SKR, Nouri M, Olive V, Kobelev M, Okumura K, McCarthy D, et al. The long noncoding RNA H19 regulates tumor plasticity in neuroendocrine prostate cancer. Nat Commun. 2021;12(1):7349. 10.1038/s41467-021-26901-9 PMC8692330 34934057

[42] Li X-J-Y, Zhou F, Li Y-J, Xue X-Y, Qu J-R, Fan G-F, Liu J, Sun R, Wu JZ, Zheng Q, et al. LncRNA H19-EZH2 interaction promotes liver fibrosis via reprogramming H3K27me3 profiles. Acta Pharmacol Sin. 2023;44(12):2479–2491. 10.1038/s41401-023-01145-z PMC10692088 37580495

[43] Yu X, Liu Q, He J, Huang Y, Jiang L, Xie X, Liu J, Chen L, Wei L, Qin Y. Vigilin interacts with CTCF and is involved in the maintenance of imprinting of IGF2 through a novel RNA-mediated mechanism. Int J Biol Macromol. 2018;108:515–522. 10.1016/j.ijbiomac.2017.11.109 29157910

[44] Yang Y, Tang F, Wei F, Yang L, Kuang C, Zhang H, Deng J, Wu Q. Silencing of long non-coding RNA H19 downregulates CTCF to protect against atherosclerosis by upregulating PKD1 expression in ApoE knockout mice. Aging. 2019;11(22):10016–10030. 10.18632/aging.102388 PMC6914395 31757932

[45] Zhang L, Yang Z, Trottier J, Barbier O, Wang L. LncRNA MEG3 induces cholestatic liver injury by interaction with PTBP1 to facilitate Shp mRNA decay. Hepatology. 2017;65(2):604–615. 10.1002/hep.28882 PMC5258819 27770549

[46] Cordaro A, Barreca MM, Zichittella C, Loria M, Anello D, Arena G, Sciaraffa N, Coronnello C, Pizzolanti G, Alessandro R, et al. Regulatory role of lncH19 in RAC1 alternative splicing: Implication for RAC1B expression in colorectal cancer. J Exp Clin Cancer Res. 2024;43(1):217. 10.1186/s13046-024-03139-z PMC11299361 39098911

[47] Wang S-H, Zhu X-L, Wang F, Chen S-X, Chen Z-T, Qiu Q, Liu WH, Wu MX, Deng BQ, Xie Y, et al. LncRNA H19 governs mitophagy and restores mitochondrial respiration in the heart through Pink1/Parkin signaling during obesity. Cell Death Dis. 2021;12(6):557. 10.1038/s41419-021-03821-6 PMC8163878 34050133

[48] Prakrithi P, Vo T, Xiong Z, Vu H, Nguyen LT, Newman A, Wilhelmey J, Whitehall V, Cruz JL, Wood S, et al. Unraveling lncRNA diversity at a single cell resolution and in a spatial context across different cancer types. Nat Methods. 2026;23:1236–1249. 10.1038/s41592-026-03071-4 PMC13259954 42135486

[49] Liu SJ, Nowakowski TJ, Pollen AA, Lui JH, Horlbeck MA, Attenello FJ, He D, Weissman JS, Kriegstein AR, Diaz AA, et al. Single-cell analysis of long non-coding RNAs in the developing human neocortex. Genome Biol. 2016;17(1):67. 10.1186/s13059-016-0932-1 PMC4831157 27081004

[50] Liu SJ, Dang HX, Lim DA, Feng FY, Maher CA. Long noncoding RNAs in cancer metastasis. Nat Rev Cancer. 2021;21(7):446–460. 10.1038/s41568-021-00353-1 PMC8288800 33953369

[51] Nemeth K, Bayraktar R, Ferracin M, Calin GA. Non-coding RNAs in disease: From mechanisms to therapeutics. Nat Rev Genet. 2024;25(3):211–232. 10.1038/s41576-023-00662-1 37968332

[52] Lin Q, Sheng J, Zhou C, Xiao T, Meng Y, Lu M, Zhang J, Yan X, Peng L, Cao H, et al. LPItabformer: Enhancing generalization in predicting lncRNA-protein interactions via a tabular transformer. Comput Struct Biotechnol J. 2025;27:2323–2335. 10.1016/j.csbj.2025.05.050 PMC12167069 40520600

[53] Florentino BR, Parmezan Bonidia R, Sanches NH, Da Rocha UN, De Carvalho ACPLF. BioPrediction-RPI: Democratizing the prediction of interaction between non-coding RNA and protein with end-to-end machine learning. Comput Struct Biotechnol J. 2024;23:2267–2276. 10.1016/j.csbj.2024.05.031 PMC11140557 38827228

[54] Zheng H, Xu L, Xie H, Xie J, Ma Y, Hu Y, Wu L, Chen J, Wang M, Yi Y, et al. RIscoper 2.0: A deep learning tool to extract RNA biomedical relation sentences from literature. Comput Struct Biotechnol J. 2024;23:1469–1476. 10.1016/j.csbj.2024.03.017 PMC11016866 38623560

