# Supplementary material for: HuRInterDB: A Database Exploring the Landscape of lncRNA–Protein Interactions
Source: Comput Struct Biotechnol J. 2026 Oct 21;35(1):0241. doi: 10.34133/csbj.0241 (PMC13683948; doi:10.34133/csbj.0241)
Supplement: Supplementary file 1 [file csbj.0241.f1.zip › Figure_S1.pdf]

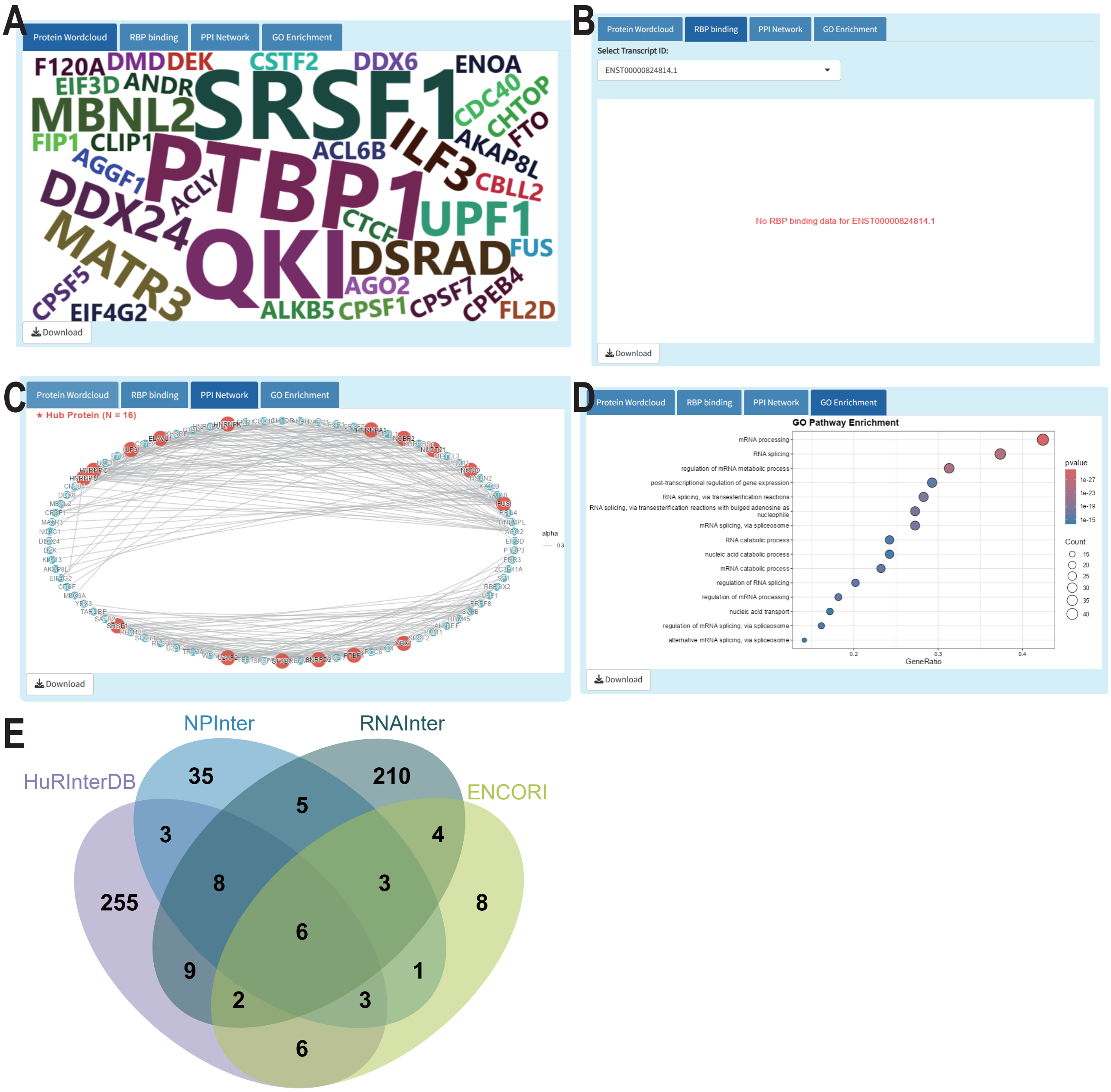
A
Protein Wordcloud
RBP binding
PPI Network
GO Enrichment
F120A DMD DEK CSTF2 DDX6 ENOA
EIF3D ANDR SRSF1 CDC40 CHTOP
MBNL2 FTO
FIP1 CLIP1 ACL6B ILF3 AKAP8L
AGGF1 PTBP1 CBLL2
DDX24 ACLY UPF1
CTCF
MATR3 QKI DSRAD FUS
CPSF5 AGO2 CPSF7 CPEB4
EIF4G2 ALKB5 CPSF1 FL2D
Download
B
Protein Wordcloud
RBP binding
PPI Network
GO Enrichment
Select Transcript ID:
ENST00000824814.1
No RBP binding data for ENST00000824814.1
Download
C
Protein Wordcloud
RBP binding
PPI Network
GO Enrichment
★ Hub Protein (N = 16)
alpha
0.3
Download
D
Protein Wordcloud
RBP binding
PPI Network
GO Enrichment
GO Pathway Enrichment
mRNA processing
RNA splicing
regulation of mRNA metabolic process
post-transcriptional regulation of gene expression
RNA splicing, via transesterification reactions
RNA splicing, via transesterification reactions with bulged adenosine as nucleophile
mRNA splicing, via spliceosome
RNA catabolic process
nucleic acid catabolic process
mRNA catabolic process
regulation of RNA splicing
regulation of mRNA processing
nucleic acid transport
regulation of mRNA splicing, via spliceosome
alternative mRNA splicing, via spliceosome
0.2
0.3
0.4
GeneRatio
pvalue
1e-27
1e-23
1e-19
1e-15
Count
15
20
25
30
35
40
Download
E
NPInter
RNAInter
HuRInterDB
ENCORI
35
210
5
3
4
8
3
255
8
6
9
1
2
3
6
