# Supplementary material for: HuRInterDB: A Database Exploring the Landscape of lncRNA–Protein Interactions
Source: Comput Struct Biotechnol J. 2026 Oct 21;35(1):0241. doi: 10.34133/csbj.0241 (PMC13683948; doi:10.34133/csbj.0241)
Supplement: Supplementary file 1 [file csbj.0241.f1.zip › Supplementary Fig1 caption.docx]

**Supplementary Fig. 1. Online analysis of MEG3–protein interactions and database coverage comparison.** (A) Word cloud of the MEG3-interacting protein; (B) Schematic plot depicting protein-binding positions across the full-length MEG3 transcript; (C) PPI network among MEG3-interacting proteins; (D) Dot plot visualizing GO enrichment results of MEG3-interacting proteins; (E) Venn diagram illustrating the overlap of H19-interacting proteins among HuRInterDB, ENCORI, NPInter, and RNAInter.
